# Facile Interfacial Engineering of Mesoporous TiO_2_ for Low-Temperature Processed Perovskite Solar Cells

**DOI:** 10.3390/nano9091220

**Published:** 2019-08-29

**Authors:** Jiyoon Nam, Inje Nam, Eun-Jin Song, Jung-Dae Kwon, Jongbok Kim, Chang Su Kim, Sungjin Jo

**Affiliations:** 1 School of Architectural, Civil, Environmental and Energy Engineering, Kyungpook National University, Daegu 41566, Korea; 2 Surface Technology Division, Korea Institute of Materials Science, 797 Changwondaero, Sungsan-Gu, Changwon, Gyeongnam 51508, Korea; 3 Department of Materials Science and Engineering, Kumoh National Institute of Technology, Gumi 39177, Korea

**Keywords:** mesoporous TiO_2_, perovskite solar cell, low-temperature processed TiO_2_, flexible solar cell

## Abstract

The mesoporous TiO_2_ nanoparticle-based scaffold structure is the best electron transport layer (ETL) for perovskite solar cells (PSCs) and is still used in most PSCs with optimal photovoltaic characteristics. However, the high sintering temperature of TiO_2_ nanoparticles required to remove binders from the TiO_2_ paste limits PSC application to flexible electronics. In this study, a simple interface modification process involving ethanol rinsing is developed to enhance the photovoltaic characteristics of low-temperature processed PSCs. This easy and fast technique could enable remarkable performance by PSCs by significantly increasing the fill factor and current density, leading to a power conversion efficiency more than four times that of untreated solar cells.

## 1. Introduction

Hybrid metal–halide perovskite solar cells (PSCs) have attracted considerable attention because of their low cost, high efficiency, and ease of fabrication. In addition to studies focused on their photovoltaic characteristics and the recent accomplishment of a power conversion efficiency (PCE) above 23% [1,2], research fields based on their flexible applications are also receiving much attention [3,4,5]. Conventional PSC fabrication methods, based on a solution process, are suitable for flexible polymer substrates [6,7]. However, the high-temperature sintering process that is required to remove organic binders and solvents from the TiO_2_ paste used for the fabrication of the mesoporous TiO_2_ electron transport layer (ETL) is not feasible for flexible polymer substrates. Hence, this high-temperature process limits the variety of substrates and the mass production scalability. To overcome these limitations, novel low-temperature processes and alternative materials for TiO_2_ ETL fabrication have been investigated.

Mesoporous TiO_2_ is still the best ETL for state-of-the-art PSCs [1,8,9,10]; the mesoporous scaffold structure is highly advantageous for electron transport since it increases the surface coverage between the TiO_2_ nanoparticles and the perovskite light-absorbing layer [11]. Furthermore, the position of the conduction band minimum of TiO_2_ is favorable for smooth electron transport from the light absorbing layer with a fast electron injection rate [12,13,14]. Nevertheless, studies on flexible PSCs based on mesoporous TiO_2_ ETLs are scarce because of the abovementioned limitations. On the other hand, the substitution of mesoporous TiO_2_ with low-temperature processable materials, such as [6,6]-phenyl-C_61_-butyric acid methyl ester (PCBM), poly(3,4-ethylenedioxythiophene)–poly(styrene sulfonate) (PEDOT:PSS), ZnO, Zn_2_SnO_4_, or Al_2_O_3_, is a common strategy to fabricate flexible PSCs [15,16,17,18]. However, the resulting photovoltaic characteristics are still inferior to those of mesoporous TiO_2_-based devices. Several research groups have proposed different deposition methods for TiO_2_ ETLs, including atomic layer deposition (ALD), electron beam deposition, and sputtering, to avoid the high-temperature sintering process. Still, all these techniques require some high-cost vacuum deposition equipment [19,20,21]. Low-temperature synthesis procedures for TiO_2_ nanoparticles have also been reported [22,23]. However, the lower electron mobility and the creation of surface charge traps hinder the charge transport at the TiO_2_/perovskite interface [24].

Here, we propose a facile interfacial engineering method to fabricate PSCs based on low-temperature sintered mesoporous TiO_2_ (LT-PSCs). We developed a novel simple surface modification process (hereafter, SMP) that involves ethanol rinsing at the end of the mesoporous TiO_2_ nanoparticles annealing; this SMP enhanced the PCE of the LT-PSCs more than four times compared with untreated PSCs. The proposed SMP has great scalability because it allows the use of conventional mesoporous TiO_2_ ETLs at low temperatures. The reported findings herein could refine the current research direction for flexible PSCs.

## 2. Materials and Methods

### 2.1. Substrate Fabrication

A fluorine-doped tin oxide (FTO) substrate (Pilkington) was cleaned using a detergent, deionized water, acetone, and ethanol; then, its surface was pretreated via ultraviolet–ozone for 15 min. Titanium diisopropoxide bis(acetylacetonate) was diluted in 1-butanol to fabricate a blocking layer that was spin-coated on the FTO substrate and dried at 125 °C for 5 min. A TiO_2_ nanoparticle paste (Greatcell Solar) was diluted in ethanol with a ratio of 1:10 (wt%) to obtain a mesoporous TiO_2_ layer that was spin-coated on the FTO substrate under the same conditions as the blocking layer and sintered at various temperatures (from 150 to 550 °C) for 1 h. For the fabrication of multilayer (ML)-TiO_2_, the TiO_2_ nanoparticle paste was diluted in ethanol at a ratio of 1:30 (wt%). The LT-TiO_2_-coated substrate was dipped in ethanol and stirred for 30 s after the mesoporous TiO_2_ sintering to remove TiO_2_ nanoparticle aggregates. The substrate was soaked in 20 mM aqueous TiCl_4_ solution at 90 °C for 15 min. The cleaned substrates were sintered for 30 min under each condition.

### 2.2. PSC Fabrication

Methylammonium iodide (MAI), synthesized by reacting methylamine and hydriodic acid at 0 °C for 2 h, was rinsed with diethyl ether and dried in an oven at 80 °C for 24 h. PbI_2_, MAI, dimethyl sulfoxide, and *N*,*N*-dimethylformamide were mixed to prepare a light-absorbing layer solution, which was spin-coated on the prepared substrate; diethyl ether was dripped on the center of the rotating substrate before the surface became hazed. An adduct solution-coated substrate was annealed first at 65 °C for 1 min and then at 100 °C for 10 min. To fabricate the hole transport layer, 2,2′,7,7′-tetrakis(*N*,*N*-di-*p*-methoxyphenylamine)-9,9′-spirobifluorene (Jilin), a lithium salt solution, 4-tert-butylpyridine, and chlorobenzene were mixed and the resulting solution was spin-coated on the MAPbI_3_ layer. A silver electrode was deposited by using a thermal evaporator. All materials and chemicals were purchased from Sigma-Aldrich.

### 2.3. Measurements

The X-ray diffraction (XRD) spectra were recorded using a diffractometer (Bruker AXS, D8-Discover, Middlesex County, MA, USA) with a Cu X-ray tube in the 10–60° 2θ range. The photovoltaic characteristics were measured under air mass 1.5 G illumination (solar simulator, Model Sol2A, Oriel, Irvine, CA, USA) at 25 °C. Thermogravimetric analysis (TGA) was performed using a Q500 system (TA Instruments, New Castle, DE, USA) with an electronic scale during target combustion up to 600 °C. The surface profiles were characterized using a scanning electron microscope (SU8220, Hitachi, Tokyo, Japan).

## 3. Results and Discussion

**Figure 1:**
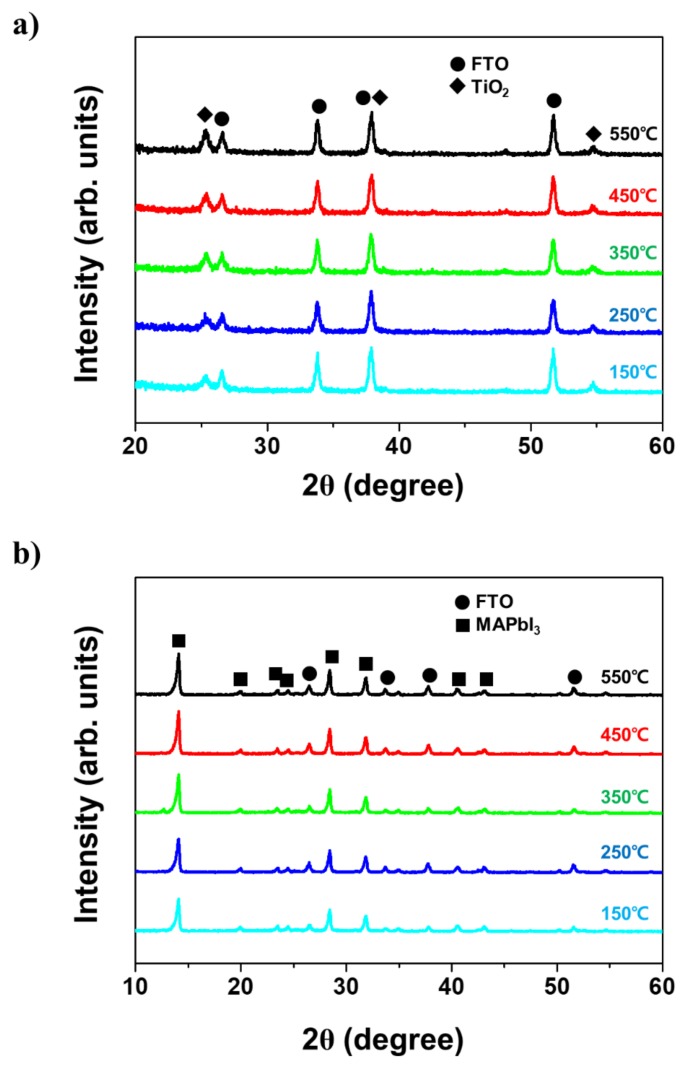
X-ray diffraction spectra of (**a**) mesoporous TiO_2_ and (**b**) CH_3_NH_3_PbI_3_ light-absorbing layers at various TiO_2_ sintering temperatures.

Residual binders resulting from the insufficient sintering temperature of mesoporous TiO_2_ paste are generally considered the reason for the degradation of the photovoltaic characteristics of PSCs [25] and, if they directly affect the solar cell properties, the crystallinity of the light-absorbing layer on TiO_2_ ETL could also be potentially altered. Therefore, we investigated X-ray diffraction (XRD) patterns of the TiO_2_ nanoparticles and the CH_3_NH_3_PbI_3_ (MAPbI_3_) light-absorbing layer at different TiO_2_ sintering temperatures to monitor their crystallinity. Figure 1a shows the XRD pattern of TiO_2_ nanoparticles at sintering temperatures between 150 and 550 °C; the diffraction peaks at 25.36°, 37.92°, and 54.82° correspond to the standard diffraction of the (101), (004), and (211) crystal planes, respectively, of anatase TiO_2_ [26]. Regardless of the sintering temperature, these diffraction peaks are consistent with the standard anatase TiO_2_ structure because commercial TiO_2_ nanoparticles dispersed in organic binders and solvents have already been manufactured in the anatase structure. As regards the light-absorbing layer (Figure 1b), the peaks observed at 14.10°, 20.00°, 23.48°, 24.46°, 28.42°, 31.86°, 40.56°, and 43.12° correspond to the diffraction of the (110), (112), (211), (202), (220), (310), (224), and (314) crystal planes, respectively, of the conventional MAPbI_3_ structure [27], and the spectra recorded at different sintering temperatures were consistent. Interestingly, these diffraction peaks did not exhibit any distinguishable phase singularity with the variation of the TiO_2_ sintering temperature.

**Figure 2:**
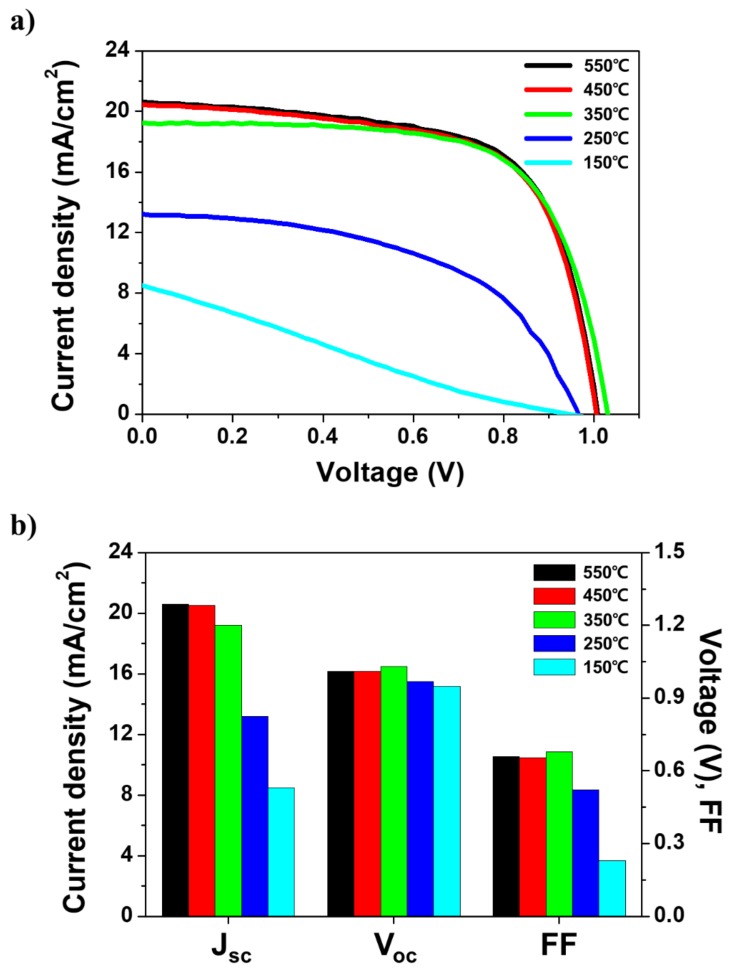
(**a**) Current–voltage curves and (**b**) variations in the current density (J_sc_), open-circuit voltage (V_oc_), and fill factor (FF) of perovskite solar cells based on mesoporous TiO_2_ sintered at different temperatures.

However, previous studies on the sintering temperature of TiO_2_ nanoparticles reported poor solar properties for PSCs fabricated at insufficient sintering temperatures [25]. We analyzed the photovoltaic characteristics of PSCs at different temperatures of mesoporous TiO_2_ sintering to distinguish the consistency following the XRD results. Figure 2 shows the resulting current–voltage curves and histograms of photovoltaic characteristics; in contrast to the XRD data, the photovoltaic characteristics degraded as the TiO_2_ sintering temperature decreased. In the 350–550 °C range, there was no significant change in the cell performance, but the photovoltaic characteristics worsened at 150 and 250 °C, indicating poor fill factor (FF) and current density (J_sc_). The photovoltaic parameters of PSCs fabricated at various TiO_2_ sintering temperatures are summarized in Table 1. The J_sc_, open-circuit voltage (V_oc_), FF, and PCE values for the high sintering temperature (550 °C) were 20.61 mA/cm^2^, 1.01 V, 0.66, and 13.67%, respectively. The PCE did not significantly degrade even when the sintering temperature was lowered to 350 °C, while J_sc_ and FF began to decrease at temperatures below 250 °C, resulting in a significantly poorer PCE (1.85%).

Despite the discordance between the solar performances estimated based on the TiO_2_ sintering temperature trend and the consistency of the XRD results, the data shown in Figure 2 indicate quite clearly the presence of certain decline points among the sintering temperatures tested. Figure 1 and Figure 2 demonstrate that other dominant factors affected the photovoltaic characteristics. Therefore, we assume that the mesoporous TiO_2_/MAPbI_3_ interface was responsible for the degradation of the photovoltaic characteristics when decreasing the sintering temperature.

**Figure 3:**
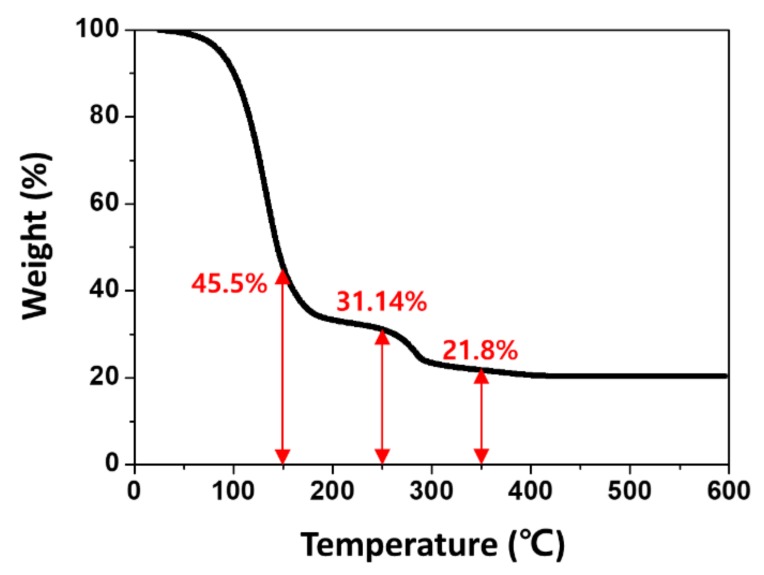
Thermogravimetric analysis curve of TiO_2_ nanoparticle paste.

Mesoporous TiO_2_ nanoparticles are generally sintered at high temperatures to remove organic binders and evaporate solvents. Since the carbonaceous residues need to be combusted at temperatures above 300 °C, the TiO_2_ sintering was conducted at 400–500 °C in most previous studies [28]. We carried out a thermogravimetric analysis (TGA) to determine the cascading tendency and verify the exact combustion or evaporation points of the binder components. TGA is usually used to identify the thermal behavior of target materials by measuring the weight of the residuals after the heating process. Figure 3 shows the temperature–weight curve for the TiO_2_ paste after annealing at 600 °C, revealing several distinct weight loss points around 100 and 300 °C. Many previous TGA studies on TiO_2_ nanoparticles have suggested various thermal behaviors for the TiO_2_ paste components [25,29]. In our data, the first weight loss at ~100 °C corresponds to the volatilization of organic solvents, which was complete at ~200 °C; the organic binders, mostly ethyl cellulose, were combusted between 200 and 300 °C, representing the second weight loss. In other words, the ETL sintered at a relatively low temperature had residues on the TiO_2_ nanoparticle surface and such interfacial impurities between the TiO_2_ ETL and MAPbI_3_ layer might have hindered the charge transport system. The small weight loss at a high sintering temperature (above 300 °C) was due to the combustion of the few carbonaceous residuals from the organic binders. Based on the TGA data, we assume that the degraded properties of the LT-PSCs were due to the insufficient organic binder combustion and solvent evaporation, which resulted in the interfacial impurities between the TiO_2_ ETL and MAPbI_3_ layer.

**Figure 4:**
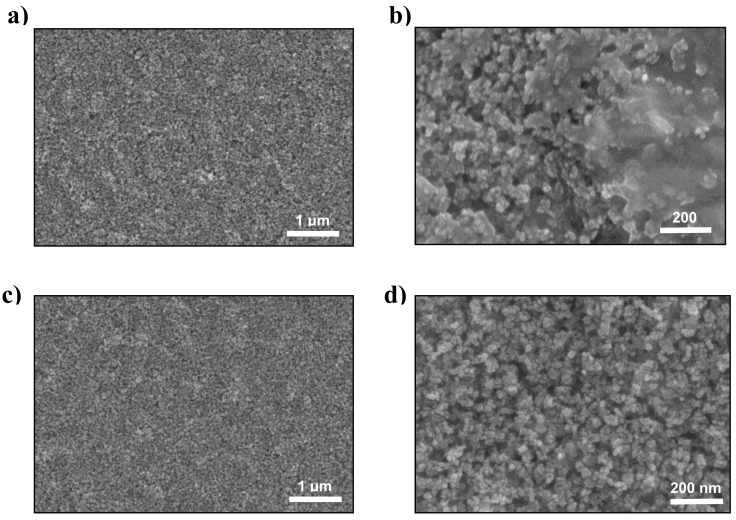
Scanning electron microscopy images of mesoporous TiO_2_ sintered at low temperatures (**a**,**b**) without and (**c**,**d**) with the surface modification process, at (**a**,**c**) 20,000× and (**b**,**d**) 100,000× magnification.

Therefore, we tried to identify other dominant factors affecting the TiO_2_ ETL surface and degrading the photovoltaic characteristics of the fabricated LT-PSCs. In our experiments, the surface coating uniformity of the MAPbI_3_ layer on the LT-TiO_2_ was inferior to that on PSCs based on high-temperature sintered mesoporous TiO_2_. The poor morphology of the MAPbI_3_ surface could be observed even with the naked eye; therefore, we collected scanning electron microscopy (SEM) images of the TiO_2_ ETL surface to microscopically determine the dominant degradation factors. Figure 4a,b displays the SEM images of the mesoporous TiO_2_ layer sintered at 150 °C without SMP, showing a typical surface profile of mesoporous scaffold at low magnification (20,000×), but also showing visible agglomerates (TiO_2_ nanoparticles and carbonaceous materials) at high magnification (100,000×). During TiCl_4_ dipping, which is an essential chemical treatment used to enhance the surface roughness and necking of mesoporous TiO_2_ [30], the agglomerates assembled on the TiO_2_ scaffold. These agglomerates are believed to hinder the formation of a uniform MAPbI_3_ layer and worsen, as interfacial defects, the surface coverage between the TiO_2_ ETL and MAPbI_3_ layer, disturbing the charge transport. Therefore, we infer that the low PCE of the fabricated LT-PSCs was caused by the presence of such agglomerates on the ETL surface. Hence, the photovoltaic characteristics should be boosted by their removal. To obtain a uniform surface of the mesoporous TiO_2_ layer, it was dipped in ethanol and stirred for 30 s after sintering at low temperature; without any further treatment, the agglomerates were entirely removed through this simple SMP (Figure 4c,d).

**Figure 5:**
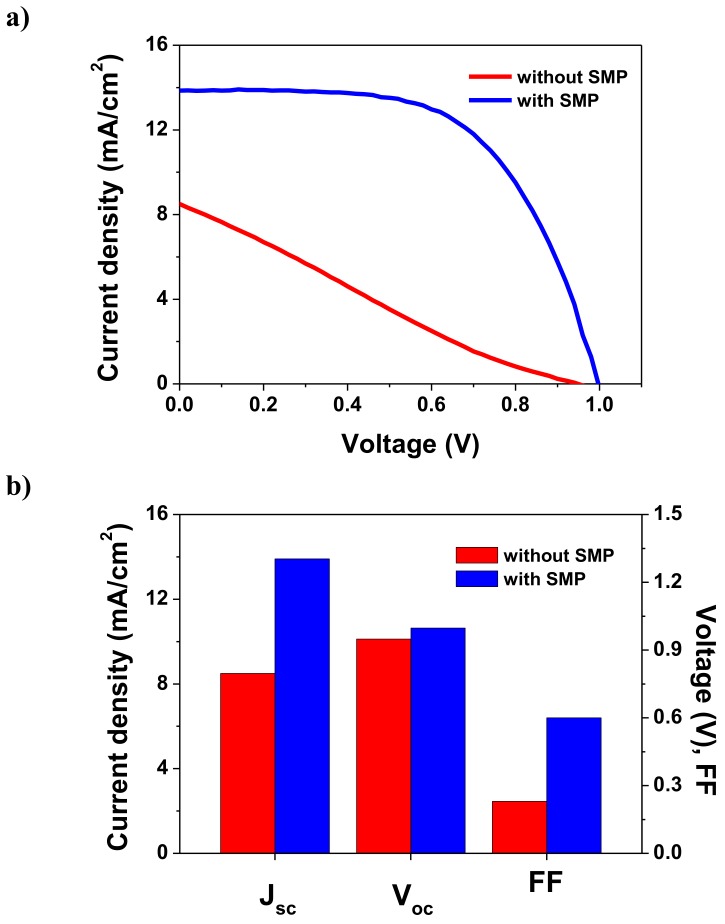
(**a**) Current–voltage curves and (**b**) current density (J_sc_), open-circuit voltage (V_oc_), and fill factor (FF) values of perovskite solar cells based on mesoporous TiO_2_ sintered at a low temperature, with and without the surface modification process (SMP).

To clarify the effectiveness of the proposed SMP, we fabricated other LT-PSCs in the same way, in addition to treating them via the SMP after the mesoporous TiO_2_ sintering at 150 °C. First, we conducted the SMP with various rinsing solvents to determine the change in the photovoltaic characteristics based on the use of different rinsing solvents such as ethanol, acetone, isopropyl alcohol (IPA), and deionized water (DI). As shown in Figure S1, solvents other than ethanol exhibited poor photovoltaic performance. It is assumed that the SMP with ethanol is the most effective method for removing the surface agglomerates because ethanol is used as the solvent for the dissolution of TiO_2_ paste to fabricate the mesoporous TiO_2_ ETL. Figure 5 compares the photovoltaic characteristics of LT-PSCs realized with and without the SMP, showing that most parameters, including the J_sc_, V_oc_, and FF, were significantly improved by the SMP (Table 1). The PCE (8.27%) was increased more than four times compared to that achieved without the SMP (1.85%), and it even exceeded that of the PSCs based on TiO_2_ sintered at 250 °C (6.63%). These results imply that the proposed SMP could enable a facile interfacial modification of low-temperature sintered TiO_2_ layers, greatly enhancing the PCE of LT-PSCs.

A multiple SMP was also developed to maximize the interface modification effect; one-third thickness of mesoporous TiO_2_ was coated and followed by SMP, and these two steps were repeated three times to complete the TiO_2_ ETL fabrication. Figure S2 shows the SEM images of the LT-TiO_2_ after a single SMP and the individual layers of multilayer TiO_2_ (ML-TiO_2_) with the SMP from the first to third layers, respectively. There were no distinguishable differences on the surface of each layer for the ML-TiO_2_ compared with the single layer of LT-TiO_2_. This corroborates the utility of the SMP, even in multiple coating processes of mesoporous TiO_2_. Furthermore, multiple iterations of the SMP improved the photovoltaic characteristics of the PSCs compared to those treated via a single SMP (Figure S3).

**Figure 6:**
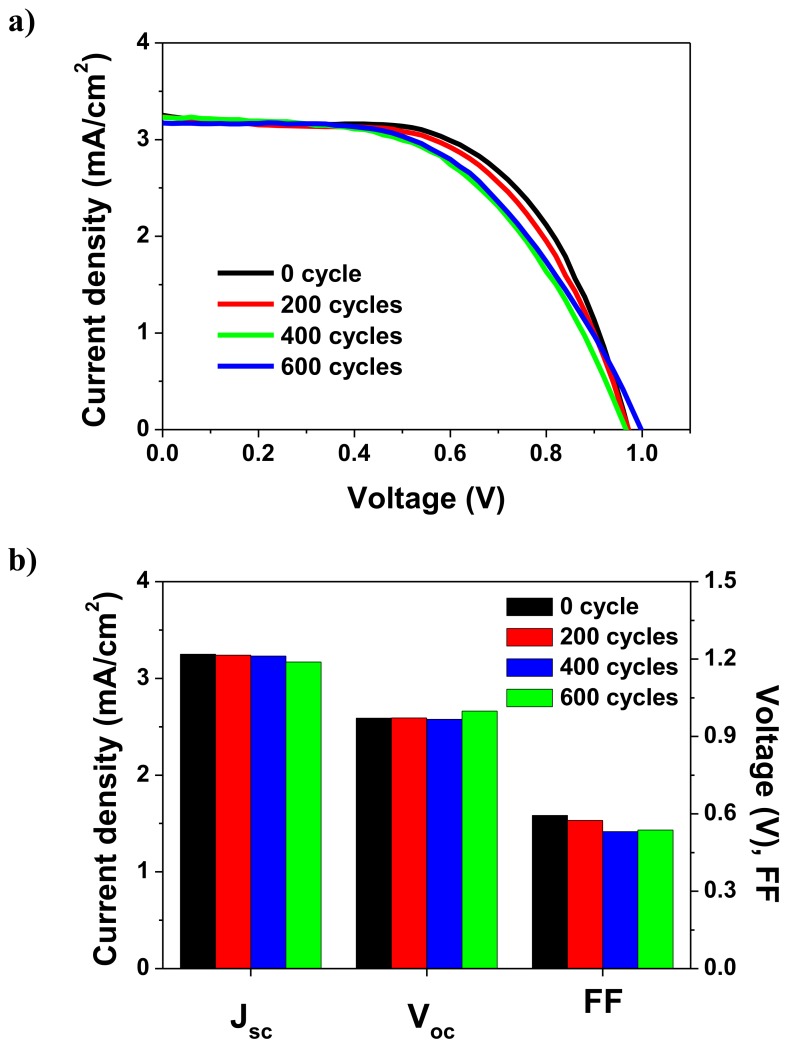
(**a**) Current–voltage curves and (**b**) variations in the current density (J_sc_), open-circuit voltage (V_oc_), and fill factor (FF) of flexible perovskite solar cells based on mesoporous TiO_2_ sintered at a low temperature and fabricated on a polyethylene naphthalate film, as functions of the bending cycles.

Finally, we successfully fabricated LT-PSCs via the SMP on a polyethylene naphthalate (PEN)/indium tin oxide (ITO) flexible substrate. The advantage of the SMP including the low-temperature process is that it facilitates the simple fabrication of flexible PSCs directly on a polymer substrate, without any substitution of the mesoporous TiO_2_ ETL. Figure 6 shows the resulting photovoltaic characteristics; these flexible LT-PSCs exhibited stable performance during the bending test at a bending radius of 10 mm.

## 4. Conclusions

The novel and simple SMP proposed in this study for LT-PSCs significantly enhanced their photovoltaic characteristics using the conventional mesoporous TiO_2_ ETL. By eliminating the agglomerates on the mesoporous TiO_2_ surface, this method guarantees a uniform and dense light-absorbing layer during the manufacturing process. The effective manufacturing of LT-PSCs was demonstrated by applying the SMP to the surface of a mesoporous TiO_2_ ETL. Significant improvements of the photovoltaic characteristics were observed. Moreover, the best ETL for PSCs and a low-temperature process could be used simultaneously. This brief and easy method to fabricate advanced LT-PSCs will benefit future developments for flexible, bendable, large-scale, and printable applications. Our ongoing research includes follow-up studies to enhance the advantages of the SMP and develop additional effective surface treatments.

## Figures and Tables

**Table 1:** Photovoltaic parameters (current density (J_sc_), open-circuit voltage (V_oc_), fill factor (FF), and power conversion efficiency (PCE)) for perovskite solar cells based on mesoporous TiO_2_ sintered at different temperatures, with and without the surface modification process (SMP).

TiO_2_ Sintering Temperature (°C)	J_sc_ (mA/cm^2^)	V_oc_ (V)	FF	PCE (%)
550	20.61	1.01	0.66	13.67
450	20.46	1.01	0.65	13.49
350	19.23	1.03	0.68	13.45
250	13.20	0.97	0.52	6.63
150 (without SMP)	8.50	0.95	0.23	1.85
150 (with SMP)	13.86	1.00	0.60	8.27

## Supplementary Materials

The following are available online at https://www.mdpi.com/2079-4991/9/9/1220/s1, Figure S1: SEM images of the surfaces of single and multilayer TiO_2_, Figure S2: Current–voltage curves and photovoltaic characteristics of perovskite solar cells based on single and multilayer TiO_2_.
