# Supplementary material for: Facile Interfacial Engineering of Mesoporous TiO2 for Low-Temperature Processed Perovskite Solar Cells
Source: Nanomaterials (Basel). 2019 Aug 29;9(9):1220. doi: 10.3390/nano9091220 (PMC6780757; doi:10.3390/nano9091220)
Supplement: Supplementary file 1 [file nanomaterials-09-01220-s001.pdf]

SUPPLEMENTARY INFORMATION

Facile Interfacial Engineering of Mesoporous TiO_2 for Low-Temperature Processed Perovskite Solar Cells

Jiyoon Nam[1], Inje Nam[1], Eun-Jin Song[2], Jung-Dae Kwon[2], Jongbok Kim[3], Chang Su Kim[2], and Sungjin Jo[1,*]

[1]School of Architectural, Civil, Environmental and Energy Engineering, Kyungpook National University, Daegu 41566, Republic of Korea

[2]Surface Technology Division, Korea Institute of Materials Science, 797 Changwondaero, Sungsan-Gu, Changwon, Gyeongnam 51508, Republic of Korea

[3]Department of Materials Science and Engineering, Kumoh National Institute of Technology, Gumi 39177, Republic of Korea

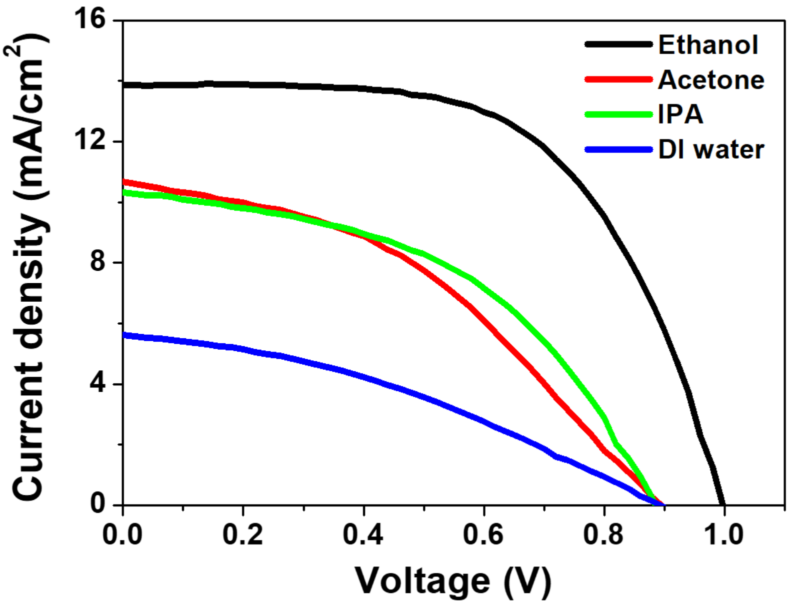

Figure S1. Current–voltage curves of perovskite solar cells based on the surface modification process (SMP) with various rinsing solvents.

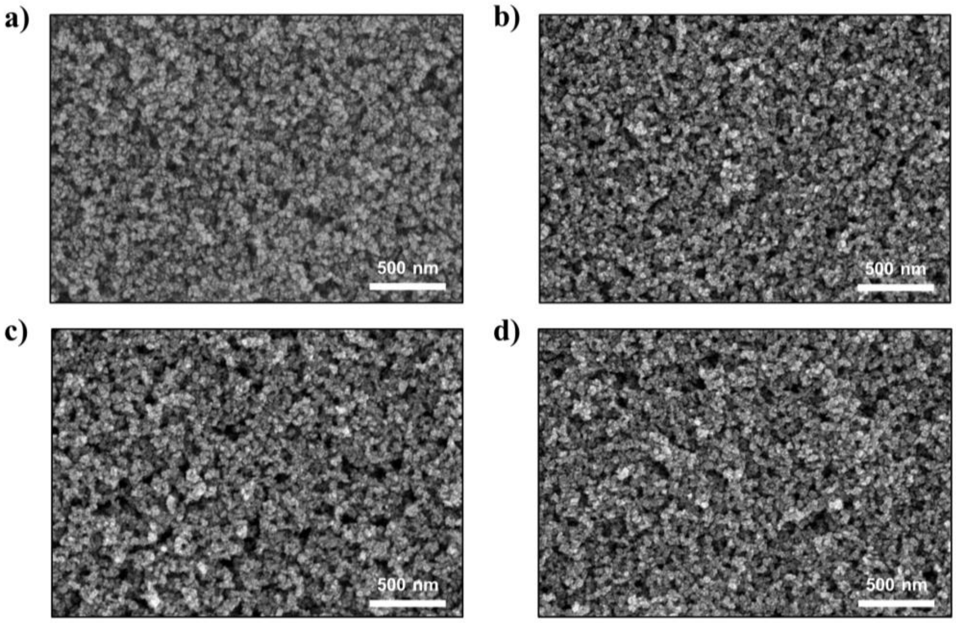

Figure S2. Scanning electron microscopy images of the surfaces of (**a**) TiO_2 sintered at a low temperature and the (**b**) first, (**c**) second, and (**d**) third layers of multilayer TiO_2.

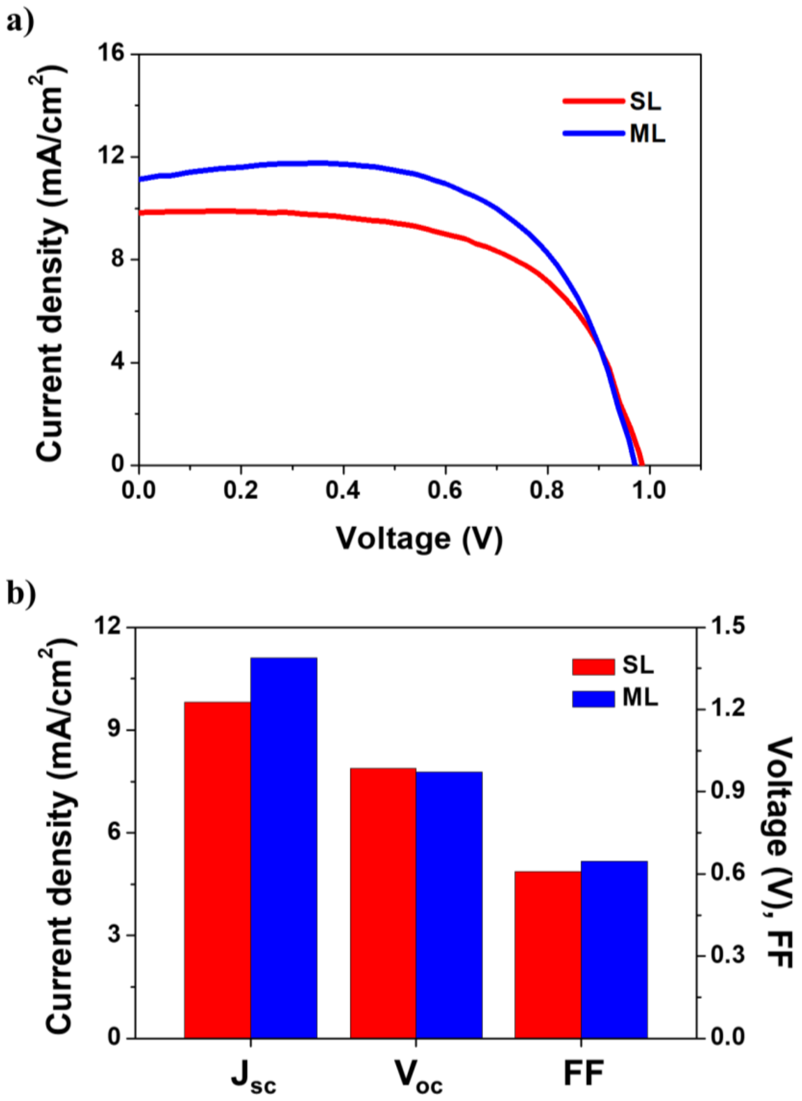

Figure S3. (**a**) Current–voltage curves and (**b**) current density (J_{sc}), open-circuit voltage (V_{oc}), and fill factor (FF) values of perovskite solar cells based on single layer (SL) and multilayer (ML) TiO_2 sintered at a low temperature.
